# Anxiety and Depressive Disorders in Subclinical vs Overt Hypothyroidism: Clinical Structure and Short-Term Dynamics

**DOI:** 10.1192/j.eurpsy.2026.11071

**Published:** 2026-07-31

**Authors:** B. Vosikov, S. Magzumova

**Affiliations:** Psychiatry and Narcology, Tashkent State Medical University, Tashkent, Uzbekistan

## Abstract

**Introduction:**

Hypothyroidism is linked to anxiety and depression, but comparative clinical profiles and short-term dynamics across subclinical and overt forms are unclear.

**Objectives:**

To compare clinical structure and short-term change of anxiety–depressive symptoms in subclinical vs overt hypothyroidism, and to assess biological, psychological, and social contributors.

**Methods:**

Single-center observational study with cross-sectional and partially prospective components. N=159 adults (20–60 y): overt hypothyroidism (OH) n=41, subclinical hypothyroidism (SH) n=68, euthyroid control with anxiety/depression (EU) n=50. Baseline and discharge assessments: HADS, HDRS-17, HARS, CGI-S/CGI-I; Schmieschek–Leonhard accentuation questionnaire. Thyroid status: TSH, free T4 (chemiluminescence). Statistics: ANOVA/Kruskal–Wallis, χ^2^/Fisher, Pearson/Spearman, multiple linear regression.

**Results:**

Symptom severity differed by group (p<0.001). OH showed highest means: HDRS-17 24.5 ± 4.2; HARS 22.8 ± 3.9; HADS-D 15.7 ± 3.1; HADS-A 13.6 ± 2.5; CGI-S median 5.0 [5–6]. SH and EU were lower yet clinically significant. Symptom structure: OH predominated in somatovegetative components; EU showed cognitive-affective predominance (p<0.01); SH was intermediate and heterogeneous. Short-term change favored OH: ΔHDRS-17 −15.2 ± 6.1; ΔHARS −13.9 ± 5.8; ΔHADS-D −9.8 ± 4.5; 71.1% achieved CGI-I 1–2. EU improved less (62.2% CGI-I 1–2). SH showed modest change; 40.5% had no improvement/worsening. Higher TSH independently predicted greater HDRS-17 severity (β=0.35; +1.8 points per +5 mIU/L; p<0.001). Anxiety, dysthymic, and emotive accentuations were more frequent in hypothyroid groups and associated with somatic anxiety and longer course. Chronic stressors and family endocrine burden were more prevalent in SH/OH vs EU and amplified symptoms.

**Conclusions:**

Anxiety–depressive disorders in hypothyroidism are multifactorial. Overt hypothyroidism presents with somatovegetative dominance and marked short-term improvement under targeted treatment, while subclinical hypothyroidism shows heterogeneous profiles with limited short-term response. Management should be stratified: prioritize timely thyroxine replacement in OH; combine endocrine optimization with psychotherapy and stress-management in SH. Findings support interdisciplinary care pathways for patients with thyroid dysfunction and comorbid affective symptoms.

**Disclosure of Interest:**

None Declared

